# Cutis verticis gyrata and Noonan syndrome: report of two cases with pathogenetic variant in SOS1 gene

**DOI:** 10.1186/s13052-022-01340-4

**Published:** 2022-08-19

**Authors:** Francesca Mercadante, Ettore Piro, Martina Busè, Emanuela Salzano, Arturo Ferrara, Gregorio Serra, Cristina Passarello, Giovanni Corsello, Maria Piccione

**Affiliations:** 1 U.O.C. Medical Genetics, AOOR Villa Sofia-Cervello, Via Trabucco, 180, 90146 Palermo, PA Italy; 2 Department of Health Promotion, Mother and Child Care, Internal Medicine and Medical Specialties, University of Palermo, Palermo, Italy; 3 Department of Services, AOOR Villa Sofia-Cervello, Palermo, Italy

**Keywords:** SOS1, K170E, Cutis verticis gyrata, Noonan syndrome, Case report

## Abstract

**Background:**

Noonan and Noonan-like syndromes are multisystem genetic disorders, mainly with autosomal dominant trasmission, caused by mutations in several genes. Missense pathogenetic variants of SOS1 gene are the second most common cause of Noonan syndrome (NS) and account approximately for 13% to 17% of cases. Subjects carrying a pathogenetic variant in SOS1 gene tend to exhibit a distinctive phenotype that is characterized by ectodermal abnormalities. *Cutis verticis gyrata* (CVG) is a rare disease, congenital or acquired, characterized by the redundancy of skin on scalp, forming thick skin folds and grooves of similar aspect to cerebral cortex gyri. Several references in the literature have reported association between nonessential primary form of CVG and NS.

**Case presentation:**

we report two cases of newborns with CVG and phenotype suggestive for NS who have been diagnosed to harbour the same pathogenetic variant in SOS1 gene.

**Conclusions:**

previously described patients with NS presenting CVG had received only clinical diagnosis. Therefore we report the first patients with CVG in which the clinical suspicion of NS is confirmed by molecolar analysis.

## Background

Noonan and Noonan-like syndromes are multisystem genetic disorders, mainly with autosomal dominant trasmission, caused by mutations in several genes: PTPN11 (OMIM 176,876), KRAS (OMIM 190,070), SOS1 (OMIM 182,530), RAF1 (OMIM 164,760), NRAS (OMIM 164,790), BRAF (OMIM 164,757), RIT1 (OMIM 609,591), SOS2 (OMIM 601,247), LZTR1 (OMIM 600,574), MRAS (OMIM 608,435), RRAS2 (OMIM 165,090), MAPK1 (OMIM 176,948), RRAS2 (OMIM 600,098), A2ML1 (OMIM 616,027), CBL (OMIM 165,360), SHOC2 (OMIM 602,775), PPP1CB (OMIM 600,590).

The PTPN11, SOS1, KRAS, RAF1, BRAF and MEK1(MAP2K1) genes, account for approximately 70% of affected individuals. SHP2 (encoded by PTPN11), SOS1, BRAF, RAF1 and MEK1 positively contribute to RAS-MAPK signaling through complex autoinhibitory mechanisms, that fail when these genes have mutated.

Whatever the altered gene is, the expressiveness of the clinical picture is variable even within the same family. Clinically NS is characterized by the presence of postnatal growth impairment with short stature, peculiar craniofacial characteristics and congenital heart disease as pulmonary valve stenosis (50–60%), hypertrophic cardiomyopathy (20%), atrial septal defects (8%), interventricular septal defects (5%). Electrocardiographic changes including left axial deviation, an abnormal R / S ratio on the left precordial leads and an abnormal Q wave, or heart rhythm disturbances in patients with hypertrophic cardiomyopathy are reported in a variable percentage of patients. Other signs frequently associated with the syndrome are pterygium colli, deformity of the rib cage, delayed psychomotor development of varying degrees, anomalies of the genitourinary system (cryptorchidism, hypospadias, micropenis), anomalies of the lymphatic system (hypoplasia of the lymphatic vessels), ocular abnormalities (strabismus, myopia), ectodermal component and dentition anomalies (pilar keratosis, skin discoloration, bristly or fluffy hair, malocclusion) and bleeding tendency for coagulation anomalies with deficiency of some coagulation factors. An increased susceptibility to develop autoimmune diseases (celiac disease, vasculitis, thyroiditis, SLE, uveitis) and myeloproliferative disorders have also been reported. [1, 36, 38, 40]

In addition to myeloproliferative disorders and acute lymphoblastic leukaemia, several solid tumors have been reported in individuals with NS, mainly embryonal rhabdomyosarcoma, neuroblastoma, and glial tumors.

Although the tumor risk in patients with related SOS1 NS was previously considered lower than in other forms linked to other genes, over the years a significant incidence of some solid tumors has been reported in these patients including embryonal rhabdomyosarcoma, Sertoli cell testis tumor, granular cell tumors of the skin and mandibular multiple giant cell lesions (MGCLs). These are benign tumor-like lesions consisting of an osteoblast-like cell population, already described in patients with NS or other RAS-opathies, whose higher incidence has been described to be linked to cases due to mutations in SOS1 [2, 7, 13, 16, 20, 24, 25, 28, 35].

The dermatological findings of RASopathies consist in pigmented lesions (café-au-lait spots, lentigines, and melanocytic lesions), ectodermal lesions (ichthyosiform manifestations, follicular hyperkeratosis, short, curly, thin hair), and hyperplasia (redundant skin, papillomatous growths). Several of these skin manifestations are associated with a specific syndromic phenotype, even if the molecular mechanism determining the association of specific tegumentary lesions with specific syndromes and specific genes is still unknown.

More in detail, the abnormalities observed in patients with NS appear to depend on the mutation responsible, indeed it was observed that hyperkeratotic skin is much more frequent in NS patients harboring *SOS1* gene mutations and generally in subjects with mutations in genes directly involved in cell proliferation kinase cascades (*SOS1*, *BRAF*, *KRAS* and *RAF1*) [3, 14, 30, 31, 37].

Pathogenetic variants of SOS1 are responsible for NS but also for hereditary gingival fibromatosis type 1, a benign overgrowth condition of the gingiva characterized by a slowly progressive, benign fibrous enlargement of keratinized gingiva. Missense mutations of this gene, as in the cases under examination, are the second most common cause of NS and account approximately for 13% to 17% of cases. Subjects carrying a pathogenetic variant in SOS1 gene tend to exhibit a distinctive phenotype that is characterized by ectodermal abnormalities (keratosis, pilaris, hyperkeratocic skin, sparse eyebrows, sparse thin and curly scalp hairs) generally not associated with cognitive deficits in place of which mood disorders such as anxiety or depression have been described along with absence of growth impairment. In some subjects, fetal macrosomia, found in the prenatal period, was not correlated with postnatal growth. Patients with SOS1-related NS display typical facial features, including macrocephaly, hypertelorism, ptosis, downslanting palpebral fissures, sparse eyebrows with keratosis pylaris, curly hair a short and broad nose with upturned tip, low-set and posteriorly angulated ears, and high forehead commonly associated with bitemporal narrowing and prominent supraorbital ridges. Among the heart defects in these subjects pulmonary stenosis, atrial and ventricular septal defects, prevail, while hypertrophic cardiomyopathy is rarer (almost 10%). Digestive disorders such as gastroesophageal reflux disease, Chiari malformation, refractive disorders and musculoskeletal pain are also reported in adolescent and / or adult subjects [5, 14, 15, 24, 27, 31, 37, 42].

SOS1 gene (OMIM 182,530) encodes the Ras-GEF protein SOS1 (SOS RAS/RAC guanine nucleotide exchange factor 1), which, once translocated on the plasma membrane, binds Ras stimulating its conversion from the inactive GDP-bound form to the active GTP-bound form [11, 24, 39].

It is a large multidomain protein characterized by an N-terminal regulatory portion including tandem histone-like folds (HF), which are followed by a Dbl-homology (DH) domain, a pleckstrin-homology (PH) domain, and a C-terminal catalytic region including the RAS exchanger motif (REM) and CDC25 domains, followed by a tail providing docking sites for adaptor proteins required for receptor anchoring. The ordinary conformation of DH and PH domains (the DH-PH unit) usually blocks allosteric Ras binding. HF also performs this function and besides stabilizes the autoinhibitory conformation of the DH-PH unit. Moreover, both HF domain and the DH-PH unit are conformationally coupled to control SOS1's recruitment to the plasma membrane. This process is a necessary step to alleviate the effect of the HF and DH domains and allow the Ras link to the allosteric site that promotes a conformational rearrangement of the CDC25 domain promoting RAS binding to the catalytic site. SOS1 disease causing variants are almost always missense changes, most of whom are found in exons coding domains HF, DH and PH. Since these stabilize SOS1 inhibited conformation, their disruption causes a release of autoinhibition and consequently a gain-of-function of SOS1 that involves an increase in the active form of Ras and greater Ras/MAPK pathway signaling [11, 24, 38, 39, 42].

## Cases presentation

### Patient 1

This patient was a first child of Caucasian, non-consanguineous and apparently healthy parents, born at 36 + 2 weeks of gestation by emergency cesarean section for an abnormal cardiotocographic tracing, after a pregnancy complicated by polyhydramnios detected in the first trimester along with gestational diabetes. At birth his weight was 3000 g (76^th^ centile, 0.72 SD), length 48 cm (22^nd^ centile, 0.22 SD) and head circumference (HC) 35 cm (93^rd^ centile, 1.49 SD) (A). He underwent neonatal resuscitation with an Apgar score 6 and 7 at 1 and 5 min, respectively, and subsequently hospitalized cause respiratory problems needing noninvasive ventilatory support for two days.

**Fig. 1:**
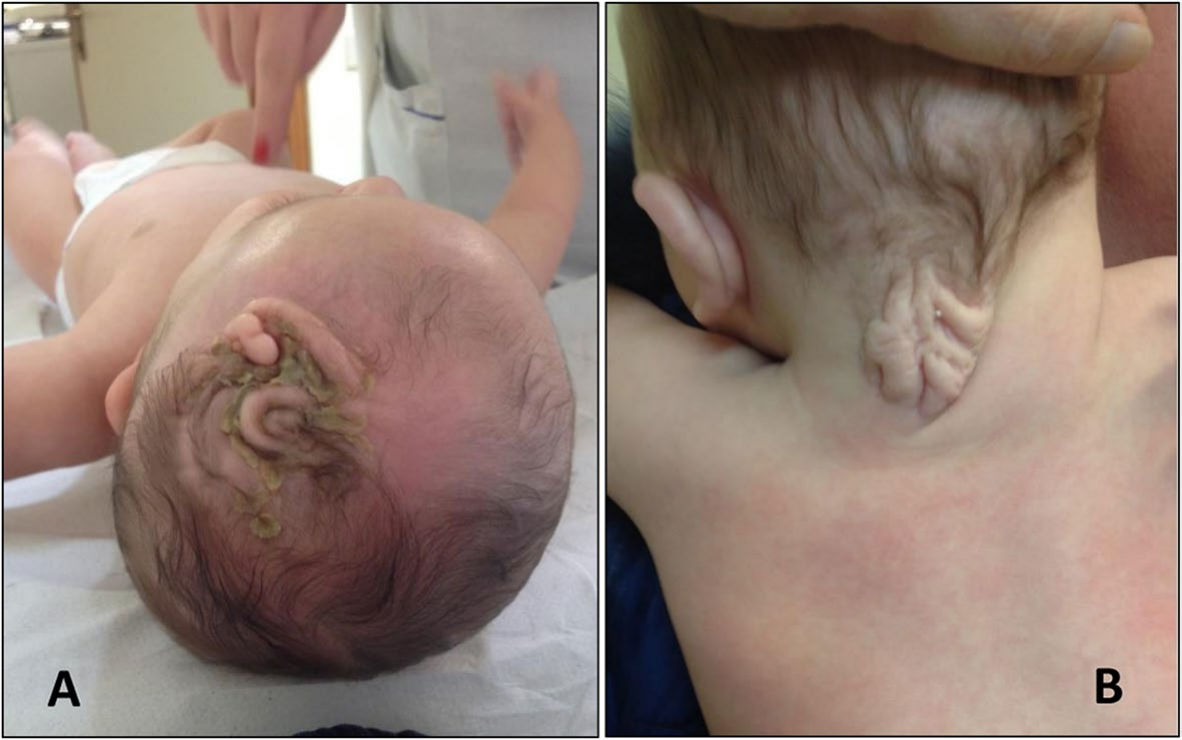
Patient 1. Cutis verticis gyrata in the frontoparietal (**A**) and nucal (**B**) region

Physical examination revealed cutis verticis gyrata in the frontoparietal and nucal regions with presence of hair in the deep furrows but not in the convoluted fold (Fig. 1), high broad forehead, hypertelorism, flat nose root, low-set, thick and posteriorly rotated ears, thin upper lip, short neck and bilateral cryptorchidism. Neurologically he showed axial hypotonia. At 2 months corrected age (CA) his dysmorphic traits were unchanged, and his growth parameters were as follows: weight 5290 g (44^th^ centile, -0.143 SD), length 56,5 cm (16^th^ centile, 0.99 SD) and HC 38,5 cm (33^rd^ centile, -0.44 SD) (B). An echocardiographic control revealed a mild pulmonary valve stenosis, and a neurodevelopmental evaluation showed a global profile adequate for the CA. Between 6 and 8 months CA an individualized follow-up included an echocardiogram that showed a patent foramen ovale with left–right shunt, mitral valve dysplasia with slight acceleration and electrocardiographic signs of mild ventricular hypertrophy. Ophthalmological evaluation showed high degree hyperopia with astigmatism and flash-visual evoked potentials showed increased P100 latencies bilaterally. At 8 months CA his HC was 40 cm (-3.7 SD) with right occipito-parietal plagiocephaly. He showed a severe central type generalized hypotonia with inability to raise the head in the prone position and to disengage the upper limbs with minimal head lifting. Spontaneous motricity was severely reduced with inability to reach the midline with his hands, to approach the rattle, eventually to hold it and no pointing. He was not able to attain the upright sitting position along with absent lateral propping bilaterally. Nevertheless, he showed an adequate reciprocal visual engagement, ability to follow the rattle, smile, environmental participation, and babbling. A brain MR showed asymmetry of the temporal ventricular horns, with dilatation of the right one and wide Sylvian fissures. In the dorso-nucal region cutis verticis gyrata appeared redundant and elongated. EEG, auditory evoked potentials, and abdominal ultrasound were normal.

### Patient 2

**Fig. 2:**
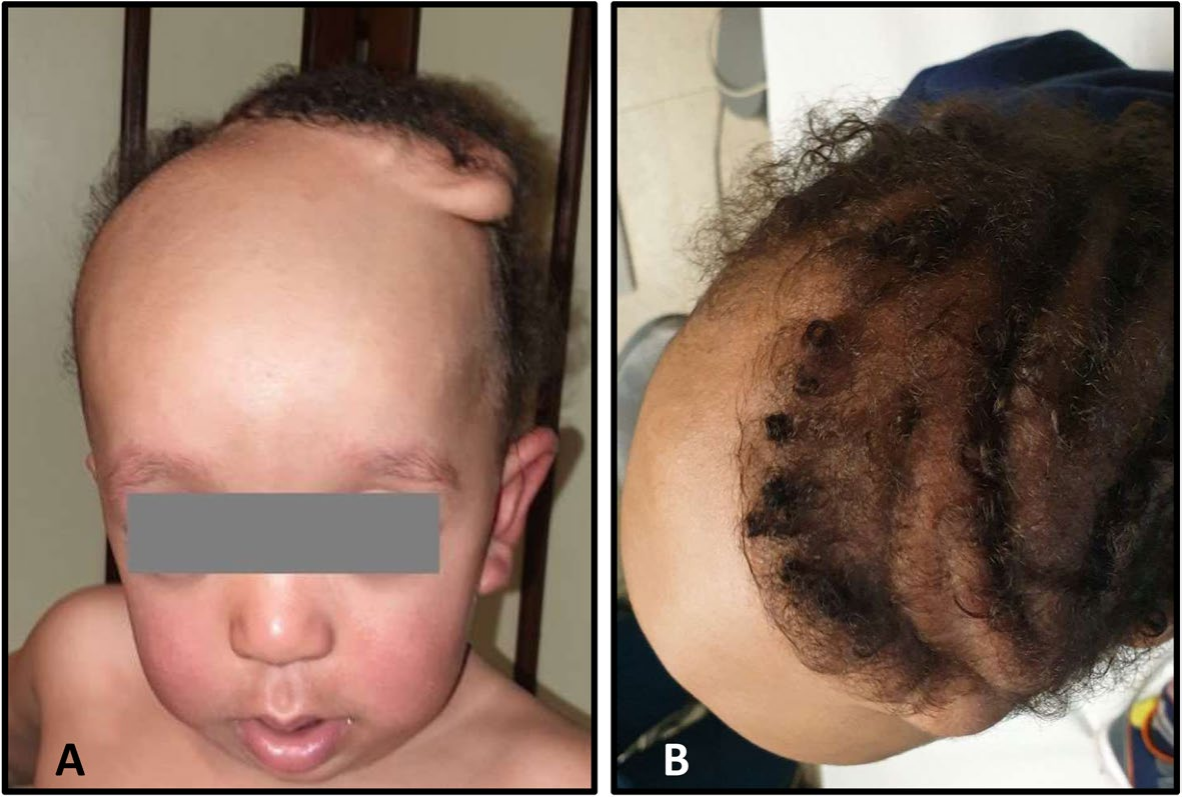
Patient 2. Front (**A**) and top (**B**) view of the cutis verticis girata

This second patient was a second child of non-consanguineous and apparently healthy Maghrebi parents, born at 38 weeks of gestation by elective cesarean section. At birth his weight was: 4500 g (3.65 SD), length 50,2 cm (67^th^ centile, 0.45 SD) and head circumference 36 cm (98^th^ centile, 2.09 SD) (A). He underwent neonatal resuscitation with an Apgar score 2 and 8 at 1 and 5 min, respectively, and subsequently hospitalized cause respiratory problems needing first invasive ventilation and then noninvasive ventilation with nasal cannulae for a total of 8 days. At 14 days of life he was transferred to our University hospital for diagnostic investigations. Physical examination revealed cutis verticis gyrata more prominent on the left side of the vertex, anteverted nostrils, cup shaped and posteriorly rotated ears, large mongolian spot in the sacral region, normal-shaped chest with teletelia, normal male genitals. Neurological examination showed a central type axial hypotonia, with poor repertoire of spontaneous motility along with weak suction. During hospitalization an echocardiogram showed a patent foramen ovale with left–right shunt and mild dysplasia and stenosis (gradient max 25 mmHg) of the pulmonary valve. Head ultrasound showed normal ultrasound appearance of the cerebral parenchyma and ventricular system with wider left frontal horn of the lateral ventricle. Blood tests, thyroid function, eye examination and ultrasound abdomen were normal. A brain MRI at 1 month of age showed widening of the periencephalic liquoral spaces and in the left hemisphere reduced development of the temporal lobe and opercular region of the frontal lobe, associated with a modest greater amplitude of the left ventricular sections compared to the right hemisphere. Incomplete rotation of the left hippocampus and hypoplastic corpus callosum, a lower insertion of the tentorium, with modest reduction in size and thickness of the worm and greater amplitude of the quadrigeminal and supravermian cisterns. A marked thickening of the subgaleal soft tissues was present in the occipital-parietal area, with greater evidence on the left side, and at the vertex. At 4 months auditory evoked potentials showed an auditory threshold of 40 dB HL in the left ear and 30 dB HL in the right one. Since an individualized follow-up showed early sings of generalized developmental delay, the infant was enrolled in a rehabilitation program. He attained the upright sitting position at 8 months, and walking at 19 months. At 29 months of age his weight was 13.800 gr (65^th^ centile, 0.39 SD), length 85 cm (3^rd^ centile, 1.92 SD) and HC 52 cm (99^th^ centile, 2.25 SD) with normocephalic parents (C) (Fig. 2). Neurodevelopmental assessment showed a mild degree of central type generalized hypotonia with clumsiness, he was able to go up and down the stairs held by the hand, he could pronounce a few two words understandable phrases and to carry out simple orders, but was unable to say its own name. The area of fine motor skills and praxis was the most appropriate for the chronological age since he was able to build a tower of eight cubes and copy the circle and straight lines. Social behavior was characterized by an initial shyness, with subsequent willingness to interact with the examiner in a pleasant and collaborative way.

### Genetic findings

Both patients underwent analysis of the main genes related to RASopathies through *Ion AmpliSeq Noonan Research Panel* (Life Technologies, Carlsbad, CA) and were found to be carriers of variant c.508 A > G, p.Lys170Glu (K170E) in the exon 4 of SOS1 gene.

This exon was subsequently sequenced using the sanger approach for both proband validation and parental segregation analysis.

In the case 1 the segregation study revealed that the variant was originated from the mother presenting only the NS peculiar facial traits, while in case 2 it showed the de novo origin of the variant.

The c.508 A > G, p.Lys170Glu (K170E) variant involves the codon 170 of SOS1 gene, residing in exon 4 that codes for the HF domain (residues 1–191). Consequently it affects the interaction between the HF, DH, and PH domains, perturbing the overall autoinhibited conformation in which the HF and DH domains block the protein, thus resulting in deleterious effect through its gain of function.

It is reported in all the databases consulted: LOVD v 3.0 (Leiden Open Variation Database, https://databases.lovd.nl/), ClinVar (http://www.ncbi.nlm.nih.gov/clinvar/), HGMD Professional 2021.4 (The Human Gene Mutation Database, ([https://portal.biobase-international.com/hgmd/pro/gene.php], /) as disease causing and according to the guidelines of the ACMG (American College of Medical Genetics) (Laboratory Practice Committee Working Group), this variant is considered to be pathogenic.

It should be emphasized that the ClinGen RASopathy Expert Panel defined the SOS1 gene to be a gene with a low rate of benign missense variants and high number of pathogenic missense variants, also highlighting that K170E variant is in a location that has been defined to be a mutational hotspot of SOS1 [10].

Moreover in vitro functional studies provide evidence that the K170E variant impacts protein, leading to gain-of-function, which is an established pathogenic mechanism in NS. Analyses of the variant protein shows increased activation of Ras in cell culture compared to wildtype, due to structural changes in the autoinhibitory HF domain [23, 34].

Finally this variant has been reported in four individuals with clinical features of Noonan syndrome, both in familiar cases and in two cases where this variant has occurred de novo [7, 19, 23, 24, 34].

## Discussion and conclusions

Normal epidermis homeostasis is maintained through the balance between the proliferation migration and differentiation processes. Ras/MAP kinase cascade is one of various signal transduction pathways.

who act mutually interconnected to preside over their regulation.

Ras signaling is necessary to maintain cells within the basal layer compartment in the proliferative, undifferentiated state and a loss of Ras function leads to a decrease in proliferative capacity and subsequent entry into the terminal differentiation pathway. Ultimately its role is to support epidermal proliferative capacity and to oppose the onset of differentiation. Indeed gain-of-function studies underlined that activation of Ras signaling in the epidermis causes hyperproliferation and inhibition of differentiation, instead its activity lack resultes in opposite effect implying hypoproliferation and induction of differentiation [6, 8, 17, 18, 25, 43].

*Cutis verticis gyrata* (CVG) is a rare disease, congenital or acquired, characterized by the redundancy of skin on scalp, forming thick skin folds and grooves of similar aspect to cerebral cortex gyri. Polan and Butterworth classified it into primary form (essential and nonessential) and secondary. Primary CVG is rare [29].

The primary essential form usually starts during or after puberty, it is rare and characterized by the absence of neurological and ophthalmological changes and by exclusion of secondary causes of the disease. The nonessential form presents association with several neurological manifestations (microcephaly and seizures, intellectual disability, cerebral palsy, epilepsy) or ophthalmological changes (cataract, strabismus, blindness, retinitis pigmentosa). The secondary form may arise from use of drugs like anabolic steroids. It may also be associated with inflammatory or neoplastic processes that cause changes in the scalp structure such as: osteoarticular diseases (pachydermoperiostosis, acromegaly), pituitary tumors, intracerebral aneurysm, tuberous sclerosis, amyloidosis, myxedema, dermatofibroma, acanthosis nigricans, acne conglobata, cerebriform intradermal nevus, cutaneous focal mucinosis, scalp psoriasis, syphilis,diabetes mellitus type 2. It has been reported a strong association between nonessential primary forms and chromosomal and genetic abnormalities, such as Noonan syndrome, Beare-Stevenson syndrome, Ehlers-Danlos Syndrome, "Michelin tire baby" syndrome, Turner syndrome and fragile X syndrome [4, 29, 32, 33, 41].

Even if the precise mechanism responsible for CVG remains to be determined, Larralde et al. proposed congenital lymphedema as a possible etiopathogenetical cause of CVG in Turner and Noonan’s syndromes, where in uterus compression may fix lymphedematous skin into the folds and therefore the subsequent resolution of lymphedema leaves redundant skin [21]. Similarly, it was considered that cystic hygroma occurs due to failure of the lymphatic vessels to mature during the intrauterine period of life and the pterygium coli may be explained by the regression of a cystic hygroma following correction of the lymphatic obstruction, or the formation of collateral lymphatic channels. The edema may also affect the migration of tissues during development of the embryo explaining the anomalous location of some structures (e.g., cryptorchidism, separated nipples, hypertelorism) and may also explain the development of pulmonary stenosis [4, 9, 21, 22]**.**

Several references in the literature have reported association between nonessential primary form of CVG and NS. Nevertheless, to our knowledge, in the previous reports the diagnosis of NS had been formulated only on the patient’s clinical features, lacking a genetic analysis to confirm the clinical suspicion [4, 9, 12, 22, 26].

A possible etiopathogenetic link between lymphedema regression, causing redundant skin, and nonessential primary form of CVG in patients with a clinical diagnosis of NS has been hypothesized by several authors [4, 9, 12, 21, 22, 26].

Besides, gain-of-function studies underlined that activation of Ras signaling in the epidermis causes hyperproliferation and inhibition of differentiation and it was observed that hyperkeratotic skin is much more frequent in NS patients harboring *SOS1* gene mutations and generally in subjects with mutations in genes directly involved in cell proliferation kinase cascades (*SOS1*, *BRAF*, *KRAS* and *RAF1*) [3, 6, 8, 17, 18, 30, 37].

The gain of function K170E variant in the SOS1 gene has been reported in several individuals with clinical features of Noonan syndrome, both family and de novo, but CVG was no present in none of them [7, 19, 23, 24, 34].

Our probands present a clinical and dysmorphological picture consistent with the diagnosis of Noonan syndrome related to pathogenic variants of the SOS1 gene and with many common elements between the two cases, including the presence of CVG. Infact they present: history of polyhydramnios in the prenatal period, characteristic facial features, hypotonus, cardiological findings and the absence of growth impairment both in the pre- and post-natal period.

Nevertheless, to our knowledge, in the previous reports on patients with NS and CVG the diagnosis of NS had been formulated only on the patient’s clinical features, lacking a genetic analysis to confirm the clinical suspicion. Therefore these are the first patients with CVG in which a genetic diagnosis of NS is reported.

## Data Availability

The datasets used and/or analyzed during the current study are available from the corresponding author on reasonable request.

## Abbreviations

NS: Noonan syndrome
CVG: Cutis Verticis Gyrata
SD: Standard Deviations
CA: Corrected Age
HC: Head Circumference
